# Editorialets

**Published:** 1907-05

**Authors:** 


### Editorialets.

---

"DON'T WORRY; DON'T FRET.—What's the use? Take the lean with the fat, and let it go at that."

---

DR. W. I. M. SMITH, of Nacogdoches, lost his office, fixtures, instruments, library and records by fire recently—a clean wipe out. He particularly regrets the loss of his files of the "Red Back."

---

DR. E. S. MCKEE, of Cincinnati, on account of his recent writings on privileged communications and professional confidence, has been made a member of the New York Medico-Legal Society and one of the editors of the *New York Medico-Legal Journal.*

---

DR. W. E. FITCH, editor Gaillard's *Southern Medicine*, New York, whose appointment as Demonstrator of Anatomy in Fordhard University Medical College we announced last month, has been promoted to Lecturer, Chair of Surgery, in that college.

---

YOUR "Red Back" comes close to being the most talked of journal in editorial circles. With regards,

Sincerely,

J. D. ALLBRIGHT,
*Allbright's Office Practitioner.*

---

EXCHANGES TAKE NOTICE.—If any exchange copy intended for this journal is still being sent to Dr. Russ, San Antonio (once associate editor), it is requested that it be sent to me at Austin, instead.

TEXAS MEDICAL JOURNAL.

---

WE ARE MUCH GRIEVED to announce the death at their home in Waco, on the 6th inst., of Mrs. Alethea Risher Halbert, wife of our esteemed friend, Dr. O. I. Halbert. The *Waco Herald* pays a beautiful tribute to this most lovable and lovely lady, and says: "In the passing of Alethea Risher Halbert, one of the strongest, sweetest notes has dropped from the music of life for those who knew and loved her."

---

Protuberant Convulsions in a Verbous Fluid.—Our observant friend Hatfield, of the *Chicago Clinic* (April number, page 132), describing an imaginary autopsy, says (on opening the skull): "We find the protuberant convulsions and the deep fissures and sulci bathed in a thick verbous fluid."

Send the specimens to Jonesey of the California tentacle—for the Council on Pharmacy. Fellow must have been taking some unethical proprietary.

---

Summer Session by the Lecturers and Assistants New Orleans Polyclinic.—This course is intended for recent graduates and other physicians who have been unable to attend earlier. It will last six weeks, and begin May 20, 1907. Teaching in sixteen branches, including the specialties, laboratory work and cadaveric operations. Table of rates: Any single branch, six weeks, $15; four weeks, $12; any two or more branches, each, six weeks, $12; four weeks, $10; all branches, six weeks, $100; four weeks, $75. For further particulars write New Orleans Polyclinic, Liberty and Tulane Avenue, New Orleans, La.

---

Texas Quarantine Inspectors.—State Health Officer Brumby has made the following three appointments of quarantine inspectors (six months' men): Dr. G. Seel, of Corpus Christi, to be stationed at Port Cavallo, near Port Lavaca; Dr. E. R. Walker, of Ballinger, to be located at Velasco, near the mouth of the Brazos river. The other six quarantine officers who were named some time ago by State Health Officer Brumby are as follows: Dr. J. H. Florence, of Brownsville, transferred to Sabine Pass; Dr. J. P. Tucker, of Galveston, at port of Galveston; Dr. P. V. Armstrong, of Dallas, at Brownsville; Dr. W. E. Lowrey, of Laredo, at Laredo; Dr. Van E. McFarland, of Eagle Pass, at Eagle Pass; Dr. P. J. Shaver, retained at El Paso.

---

Dr. Edw. C. Register's New Book.—"Practical Fever Nursing" will soon be issued from the presses of the W. B. Saunders Co. of Philadelphia. Dr. Register is well known as the editor of the Charlotte *Medical Journal* and as Professor of the Practice of Medicine in the North Carolina Medical College at Charlotte, N. C. He is a widely traveled, well-read, and a dignified, polished gentleman. He has always enjoyed a large and lucrative practice in his home city as well as in nearby towns and in adjoining States. From his varied and ripe experience in the profession, the doctor is in a position to write authoritively on the subject of "Fever Nursing."—*Gaillard's Southern Medicine.*

---

Jonesey up Against It.—It seems that somebody really does take Phil—Mill—This—here—Jones seriously, if I don't. I regard him as a joke. Not so the College of Physicians and Surgeons, San Francisco. The *N. Y. Medical Record,* May 18th, says:

A Suit for Slander.—The *Pacific Medical Journal* states that the College of Physicians and Surgeons of San Francisco has entered suit for $75,000 damages against Dr. Dudley Tait, former President of the Board of Medical Examiners, and Dr. Philip Mills Jones, editor of the *California State Journal of Medicine,* for *slander and libel.*

No joke, this. Shot his mouth off once too many.

